# The Effects of Social vs. Individual Housing of Zebrafish on Whole-Body Cortisol and Behavior in Two Tests of Anxiety

**DOI:** 10.3389/fvets.2022.859848

**Published:** 2022-03-31

**Authors:** Tuva Onarheim, Andrew M. Janczak, Janicke Nordgreen

**Affiliations:** ^1^Department of Production Animal Clinical Sciences, Faculty of Veterinary Medicine, Norwegian University of Life Science, Oslo, Norway; ^2^Department of Paraclinical Sciences, Faculty of Veterinary Medicine, Norwegian University of Life Science, Oslo, Norway

**Keywords:** cortisol, anxiety, social isolation, zebrafish, novel tank-diving test, black-white preference test

## Abstract

Two of the most used models of anxiety in zebrafish research, the novel tank-diving test (NTDT) and the black-white preference test (BWPT), are modifications of assays used in rodent research (open field test and light/dark test). There has been a thorough validation of these tests in rodents, but a similar level of knowledge is still missing in zebrafish. Adult zebrafish naturally live in shoals with conspecifics, and group housing is therefore assumed to be the optimal housing condition for zebrafish, as it allows for shoaling behavior. This study investigated how housing in social isolation affected whole-body cortisol and the behavioral responses in the NTDT and BWPT. We also examined the correlation between the behavioral responses in the two behavioral tests. We found that zebrafish housed in groups had significantly higher whole-body cortisol than individually housed zebrafish (F_1, 85_ = 25.51, *P* < 0.0001). Regardless of treatment, all groups had a general preference for the lower compartment in the NTDT and the black compartment in the BWPT. Individually housed zebrafish had a higher total number of entries to the white compartment in BWPT compared to group housed zebrafish when their first test was BWPT (F_1, 48_ = 5.79, *P* = 0.0201), but not when BWPT was their second behavioral test. Fish that had higher whole-body cortisol had a tendency toward fewer entries into the white compartment the first 3 min of the BWPT (F_1, 48_ = 3.90, *P* = 0.0540). There was no effect of housing on the behaviors registered in the NTDT. There was a positive correlation (correlation coefficient 0.40; *p* = 0.003) between transitions from black to white compartment in BWPT and transitions from lower to upper compartment in NTDT, but we did not find any association between duration in white compartment in BWPT and upper compartment in NTDT. Considering this, we suggest that further model validation is needed.

## Introduction

Housing conditions might influence zebrafish welfare, and unfavorable housing conditions may potentially influence the behavior and physiology of zebrafish, which may affect experimental results.

Zebrafish live naturally in shoals with conspecifics (1). In nature, shoaling ensures benefits such as protection from predators (2), and enhances foraging (3). Research has shown that adult zebrafish, along with several other species of fish, readily form shoals with conspecifics in research facilities (4). Group housing is therefore assumed to be the optimal housing condition for zebrafish, as it allows for shoaling behavior. Individual housing of the experimental animals might be preferable for some experimental designs and can allow for a reduction in the number of animals used in an experiment, as each housing unit only contributes one independent observation, regardless of the number of fish in that unit. However, due to the gregarious nature of zebrafish, it is reason to believe that individual housing may be stressful and leave basic behavioral needs unmet.

Conflicting results have been reported on the effect of social isolation on cortisol levels in zebrafish and other fish species used in research. While Forsatkar et al. (5) did not find any difference in cortisol after social isolation, other studies have found a lower whole-body cortisol concentration in socially isolated fish compared to group housed fish (6–8). Ramsay et al. (9) concluded that whole-body cortisol increased because of crowding, with a considerably higher cortisol level in the high-density housed fish compared to the low density housed fish.

Various paradigms have been developed to examine anxiety-like behaviors in zebrafish and other teleosts (10–12). The anxiety paradigms are commonly used in fields such as psychopharmacology and neuroscience. It is important to know to which degree social vs. individual housing influences the outcome of the tests, and whether tests believed to measure the same traits, such as anxiety, give corresponding results. Among anxiety paradigms the exploration-avoidance models are used intensively (13–16). The models are thought to expose the zebrafish to a motivational conflict in the exploration-avoidance dimension. The novel tank-diving test (NTDT), the most used anxiety paradigm in zebrafish, is a modification of the open field test used in rodents. The NTDT evokes a conflict between vertical explorative behavior and bottom-dwelling behavior. The bottom-dwelling behavior is thought to be a protective or antipredator behavior, potentially against aerial and/or sea predators. As the fish habituate to the NTDT, the fish will gradually explore the upper compartment of the test tank. The time spent in the bottom part of the test tank and the number of transitions to upper compartment, are therefore thought to measure the level of anxiety in zebrafish (17). The black-white preference test (BWPT) in zebrafish is a modification of the light/dark test in rodents. Zebrafish seem to spend more time in the black compartment, as do rodents (18). Like the bottom-dwelling in NTDT, the scototaxis behavior is also thought to be a protective behavior that will avoid detection by predators. The zebrafish is exposed to a motivational conflict between exploring the white area and seeking protection in the dark compartment. There are some conflicting results in the black preference between studies, and the black preference seems sensitive to test area set up. Champagne et al. (12) found in their study an opposite preference, a preference for the light compartment. Their apparatus contained transparent walls compared to white walls used in previous studies where the fish showed scototaxis behavior (19, 20). Cordova et al. (21) found alteration in preference depending on column depth and illumination. Facciol et al. (22) found a preference for black vs. white, but there was no preference between sides with different illuminations. Overall, the time spent in the black compartment and the number of transitions to the white compartment are thought to measure the level of anxiety in zebrafish (23).

While the tests described above are commonly used, there is a level of uncertainty to whether BWPT and NTDT exhibit similar motivational conflicts and/or produce the same level of anxiety in zebrafish (24, 25). Anxiety should be a consistent trait across anxiety-inducing situations. Moreover, behaviors meant to measure anxiety should be well correlated between models of anxiety. As both assays are meant to induce a conflict between exploring the white/upper compartment and seeking protection in the black/lower compartment, a consistent anxiety trait should result in an association in duration and entries between the two tests, given that these behaviors are valid measures of anxiety. Our prediction was that anxious zebrafish would have fewer entries and spend less time in the white/upper compartment, and present with higher cortisol levels.

Due to the importance of housing for animal welfare, the increasing use of behavioral tests, and the sensitivity of those tests to external factors, the main aim of this study was to test how social isolation affects whole-body cortisol and the behavioral responses in the novel tank-diving test and the black/white preference test. The second aim was to investigate the association between the behavioral responses in the two tests.

## Materials and Methods

### Subjects and Housing

A total of 152 adult wild-type zebrafish (*Danio rerio*) of both sexes were purchased from the experimental biomedicine unit at the faculty of Veterinary Medicine, Norwegian University of Life Sciences. The fish were brought into the lab in transportation tanks filled with water from their home tanks. The transport took <2 min. Twelve fish were immediately humanely killed as described in Section 1.2.3 before transport for cortisol analysis (see *cortisol analysis*). The 140 remaining fish were divided equally between two tanks (50 × 26 × 32 cm^3^), with a density of <2 fish per liter. The stock was left for 2 weeks to acclimatize to the new environment.

The housing system consisted of 18 fish tanks (length × depth × height; 50 × 26 × 32 cm^3^) distributed on three tiers and run on a semi-closed system. Both the fresh water that was added to the system and the water that was recirculated went through mechanical filters, carbon filters, and a UV-sterilizer. The water exchange rate was set to 2% exchange per day. Each tank had 10 brightly colored marbles and one plastic cup as environmental enrichment. Three of the four walls were covered with blue cardboard, preventing visual contact between fish in different tanks. The tank wall facing the room was left uncovered.

The fish were hand fed three times a day (09:00, 12:00, and 15:00) with live brine shrimps (Artemia) in the morning and afternoon, and dry food (SDS 300 scientific fish food) in the middle of the day. The light-dark schedule was set to 14:10 h with light turned on at 08:00. The water temperature was controlled twice a day and varied from 25.9 to 29.5°C (~27.9°C). The water quality was controlled daily and included measurements of pH, GH, KH, and nitrogen compounds (GH mean: 4.9, KH mean: 2.4, pH: 8, ${\text{NO}}_{2}^{-}$: <0.3 ppm, ${\text{NO}}_{3}^{-}$: 0-12.5 ppm, NH_3_ /${\text{NH}}_{4}^{-}$: 0 ppm).

### Treatment Groups

The experiment was carried out in six blocks from May to July 2017. There were 91 fish randomly allocated to one of two treatments; group housing (GH; *n* = 50) or individual housing (IH; *n* = 41). The GH-fish were placed in schools with 16–19 other zebrafish from the stock in a similar tank (50 × 26 × 32 cm^3^) resulting in groups of 17 to 20 zebrafish per tank (mean group size: 19, SD = 1.73). The IH-fish were moved to a tank (50 × 26 × 32 cm^3^) without conspecifics. Both groups were then left for 5 days in their new housing conditions. On the 6th day, 22 GH-fish and 13 IH-fish were randomly selected for cortisol analysis (group housing cortisol, GHC; individual housing cortisol, IHC). The remaining 56 fish were used for behavioral testing. The treatment time (days in isolation or in group housing) in our study was chosen to allow the fish some time to acclimatize to their group and tank, while also allowing one block to be tested within 1 week. After each block, the remaining fish in each GH-tank were transferred back to the stock tanks and were then used to form new groups for the GH fish in the next block. This was done to avoid the GH fish forming stable groups that would need to be replenished after each testing with two fish that would then have to settle into the group. In this way, we avoided having to use new fish for each GH group and block and could thus reduce the total number of fish used.

### Behavioral Testing

28 GH-fish and 28 IH-fish were tested in both the novel tank-diving test (NTDT) and black/white preference test (BWPT). The order of the two behavioral tests was balanced within each treatment group, giving a total of four groups; group housed fish with NTDT as the first behavioral test (GH-NTDT), group housed fish with BWPT as the first behavioral test (GH-BWPT), individually housed fish with NTDT as the first behavioral test (IH-NTDT) and individually housed fish with BWPT as the first behavioral test (IH-BWPT). All fish were tested between 10am and 4pm. The test order of the fish was randomized for each test day to prevent effects of day variations on cortisol and behavior. To transfer the fish between tanks, the fish were caught gently with a net and transported in a box filled with water. The fish were thus not lifted into air. Once a fish had completed both behavioral tests, it was humanely killed for cortisol analysis. All fish were humanely killed by placing them in an anesthetic bath with MS-222 (tricaine) in the tank water to a concentration of 200 mg tricaine per liter water. The tricaine solution was buffered to pH 7.0. After the fish had lost equilibrium and did not respond to touch, the spinal cord was cut with a scalpel and the fish placed on ice until transportation to a −80° freezer. The fish were stored at −80° until cortisol extraction.

#### Novel Tank Diving Test

##### Test Apparatus

**Figure 1:**
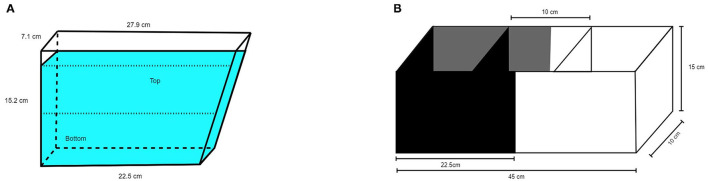
Schematic illustration of **(A)** NTDT and **(B)** BWPT.

A 1.5L trapezoidal tank (15.2 cm height, 7.1 cm width, 22.5 cm bottom, 27.9 cm top, see Figure 1A) was filled with 1350 ml home tank water to ensure that water temperature and quality was similar to the home environment. The tank was filled immediately before the first trial and the water was replaced with fresh home tank water between fish. Three of the four walls and the table the tank was standing on were covered with gray cardboard. The fourth wall was transparent to enable video recording. Measured light intensity was 85 lux. The test tank was divided into two equally high vertical zones, *upper compartment* and *lower compartment*. A video camera (Panasonic wv-CP500/G color CCTV camera) was placed 22 cm from the transparent wall.

##### Behavioral Registrations

Video recordings started immediately after the fish were placed in the test apparatus, and the recording continued for 6 min. Ethovision XT (Noldus, Wageningen, The Netherlands) was used for tracking and behavioral registrations. The following behavioral variables were registered by the software: the total number of entries to the upper compartment of the tank (Entries_NTDT_, n), total duration in the upper compartment of the tank (Duration_NTDT_, s), and latency to first transition from lower to upper compartment (Latency, s). If the fish were in the upper compartment when NTDT started, the latency was given the value 0. If the fish did not enter the upper compartment, the latency was 360 s (total time of the NTDT). All trial recordings were controlled for episodes with track errors of the zebrafish during the test. Duration, entries, and latency were manually scored by the same researcher if track errors were detected.

#### Black/White Preference Test

##### Test Apparatus

The black/white tank (Noldus, Wageningen, The Netherlands) was as described by Maximino et al. (23) with minor modifications. The tank was divided into two equally large compartments 10 × 22.5 × 15 cm^3^, one with black walls, *black compartment (BC)*, and one with white walls, *white compartment (WC)*. The full dimensions were 10 × 45 × 15 cm^3^ (width × length × height, see Figure 1B). The floor was gray in both compartments to facilitate video recording. Two sliding doors (black in the black compartment, white in the white compartment) with the dimensions 10 × 15 cm^2^ were positioned 17.5 cm from the short end of the tank, defining a central compartment (*start box*) with the dimensions 10 × 10 × 15 cm^3^. The water depth was 10 cm. The test tank water was changed between fish as described for the novel tank test. A video camera (Panasonic wv-CP500/G color CCTV camera) was placed 95 cm above the test apparatus. Measured light intensity was 77 lux in the black compartment and 83 lux in the white compartment.

##### Behavioral Registration

The fish were gently placed in the starting box and were left for 5 min to acclimatize before both sliding doors were removed. The fish's first choice of compartment (WC or BC) was registered. Video recordings started 30 s after the doors were removed and continued for 15 min. In addition to first *choice*, the following behavioral measures were included: Number of entries to the white compartment the first 3 min of the test and for the total 15 min (Entries_3min_ and Entries_total_, n) and duration of time spent in the white compartment the first 3 min of the test and for the total 15 min (Duration_3min_ and Duration_total_, s). Both entries and duration were manually scored from the video recording. The behaviors were registered if the center-point of the fish crossed the line between the BC and WC.

### Cortisol Analysis

A total of 103 zebrafish, belonging to the 7 groups listed in Table 1, were used for cortisol analysis.

**Table 1:** Schematic description of groups in this study.

Stock cortisol (SC), *n* = 12	Fish belonging to the stock purchased for this experiment. The fish were humanely killed before transfer to the research facility.
Group housing cortisol (GHC), *n* = 22	Group housed fish not subjected to behavioral testing.
Individual housing cortisol (IHC), *n* = 13	Individually housed fish not subjected to behavioral testing.
GH-BWPT, *n* = 14	Group housed fish humanely killed immediately after behavioral testing. Tested in BWPT before NTDT.
GH-NTDT, *n* = 14	Group housed fish humanely killed immediately after behavioral testing. Tested in NTDT before BWPT.
IH-BWPT, *n* = 14	Individually housed fish humanely killed immediately after behavioral testing. Tested in BWPT before NTDT.
IH-NTDT, *n* = 14	Individually housed fish humanely killed immediately after behavioral testing. Tested in NTDT before BWPT.

*N, number of fish*.

#### Cortisol Extraction

Each fish was stored in a 5 mL Eppendorf tube on ice. The fish were weighted before adding 750 uL MQ water. The fish were cut in small pieces with a scissor before homogenization. The Eppendorf tube was placed in ice water during homogenization to prevent heat degradation of cortisol. After the homogenization, the probe was rinsed with 250 uL MQ water into the Eppendorf tube. The content was then transferred into a 20 mL tube. The Eppendorf tube was rinsed twice with 250 uL MQ water that was poured into the 20 ml tube resulting in a total volume of 1500 uL MQ water added. The homogenized sample was placed on ice before transportation to a −20° freezer for storing until further preparation for LC-MS-MS. Sample sizes varied from 0.12–0.5 g tissue extract.

The LC-MS/MS method was optimized to provide a limit of quantification (LOQ) at 0.05 ng/mL and a limit of detection (LOD) at 0.015 ng/mL. The concentration of cortisol in each sample was calculated by an internal standard method using the peak area ratio and linear regression analysis. The response for cortisol was linear and gave correlation coefficients (R2) of 0.99 or better. LOD was based on 3 × signal-to-noise ratio (S/N) and LOQ was based on 10 × S/N.

On the day of LC-MS/MS analysis, the samples were thawed on ice. Cortisol and the added internal standard (cortisol-d4) were extracted with 5 mL of MTBE (methyl tert-butyl ether) from each sample in the following manner: The mixture was vortexed for 15 min, and then centrifuged at 3000 rpm for 20 min. The upper organic layer was transferred into a new 15-mL polypropylene (PP) centrifuge tube and evaporated to dryness under a gentle stream of nitrogen gas in a water bath at 25°C.

The dry residue was reconstituted in 100 μL methanol, then in 100 μL of MilliQ water (18.2Ω, Merck Millipore, Bedford, MA), filtered with Spin-X centrifuge tube filter, 0.22 μm (Costar, Utah) and transferred to a HPLC vial with insert (Agilent, Santa Clara, CA).

The HPLC-ESI-MS/MS system was performed using an Agilent 1100 setup consisting of a binary pump, degasser, and autosampler thermostat (Agilent Technologies, Santa Clara, CA) coupled to an API 4000 triple-quadrupole mass spectrometer (AB Sciex, Ontario, Canada) equipped with Turbo Ion Spray. The temperature of the autosampler was set at 5°C. Chromatographic separation was carried out on a reversed phase Kinetex C18 column, 100 × 2.1 mm, 2.6 μm particles (Phenomenex, California) with a C18 guard column in front of the column. The injection volume was 20 μL. The mobile phase consisted of 0.1% formic acid in water (A) and acetonitrile (B). A linear gradient from 15–85% B at a flow rate of 250 μL/min was used in a total 20-min runtime.

The separated compounds were detected in negative ionization-multiple reaction monitoring (MRM) mode using the respective [M+HCOO]^−^ ions, selecting one precursor ion to two products ion transitions for each compound. The mass transitions were: Cortisol m/z 407/331 as a quantifier ion transition and cortisol m/z 407/297 as a qualifier ion transition; internal standard cortisol –d4:m/z 411/335 and m/z 411/128, respectively.

The software used for controlling this equipment and acquiring and processing the data was Analyst Version 1.7 (AB Sciex, Ontario, Canada). The instrument response and the compounds parameters (declustering potential and collision energies) were optimized by using a syringe pump (Hamilton, United States) infusion of cortisol and cortisol-d4 in mobile phase at a constant flow of 10 μL/min.

The ESI source conditions of the mass spectrometer were: Collision gas (CAD): 4 psi, curtain gas (CUR): 10psi, nebulizer gas (gas 1): 50psi, turbo gas (gas 2): 50 psi, source temperature (TEM): 300°C; ion spray voltage (IS):−4400 V, entrance potential (EP): 10 V.

Possible matrix effects were determined by comparing data from calibration curves in diluent to matrix-matched ones in a range from 0.5 to 30 ng/mL for cortisol and cortisol-d4.

There were no significant matrix effects, so a standard calibration curve was used.

Calibrator's standards were prepared by dilution of the working cortisol solutions with 50% methanol to concentrations of 0.5, 1.0, 2.5, 5.0, 10.0, 15.0, and 30.0 ng/mL and 20ng/mL cortisol-d4 corresponding to 0.5 g in homogenized fish. When lower amounts of samples were used, the volume of cortisol-d4 was adapted accordingly. Two QC samples spiked with 5 ng/mL for each analysis were prepared in the same manner as an additional check of precision and accuracy of the method.

Calibration curves and negative control samples were prepared freshly for each quantitative assay.

The precision and accuracy for this method were determined by analyzing 6 replicates of the QC samples spiked at the same level (2.5 ng/mL) (RSD <9%).

All chemicals were of at least HPLC grade and supplied by Rathburn (Walkerburn, Scotland) and VWR International (Fontenay sous Bois, France). Purified water (18.2 MΩ) was obtained from a Milli-Q water purifying system from Merck Millipore (Bedford, MA). Cortisol and cortisol-d4 (certified reference standards) were purchased from Cerilliant (Sigma Aldrich, Darmstat, Germany).

### Statistical Methods

Statistical analysis was carried out in the statistical software JMP, version 14.0.0 (SAS Institute Inc., Cary, NC). Two-way ANOVA was used to analyze all data when the general linear model (GLM) requirements (normality of residuals, homogeneity of variance, and linearity) were met. Unless otherwise mentioned, the post-test was the Tukey HSD test, and the values presented are least square (LS) means. If GLM-criteria were not met, the data underwent transformation to fulfill the criteria or a non-parametric hypothesis test was conducted. Results were accepted as significant if *p* < 0.05. For student *t*-tests and non-parametric hypothesis tests, mean and SD are presented. Two fish were extreme outliers in their cortisol levels and were excluded from the study.

#### Cortisol Analysis

The effect of housing on whole-body cortisol was analyzed with ANOVA. Cortisol was transformed using square root transformation. Only housing was included as a fixed factor (model: cortisol = housing), and the analysis was performed on housing groups SC, GHC, and IHC (see Table 1).

The effect of behavior tests on cortisol was analyzed using ANOVA. Housing (treatment), behavioral tests (whether the fish went through the behavioral tests) and their interaction were included as fixed factors (model: cortisol = housing + ±behavioral tests + housing × ±behavioral tests). The analysis was performed on all groups in Table 1, except group SC. Cortisol was transformed using log transformation. For post-tests of the factors housing and behavioral tests, student *t*-test was used.

#### Behavioral Analysis

The general preference for black vs. white side in BWPT and lower vs. upper compartment in NTDT was tested using student *t*-test. Hypothesized mean, namely no compartment preference, was set to half of the total test duration for both tests and compared to the observed average duration.

Duration and entries in both NTDT and BWPT were analyzed using ANOVA with treatment, test order, and cortisol as fixed factors. Furthermore, treatment by test order and treatment by cortisol interactions were included (model: y = treatment + test order + cortisol + treatment × test order + treatment × cortisol). The student *t*-test was used as post-test. Bonferroni correction was done if there were multiple comparisons with *t*-tests. Latency to first enter the upper portion in the NTDT (*Latency*) was analyzed using Wilcoxon signed-rank test. Survival analysis, the analysis often applied if the test parameter can reach a cut-off value, was not chosen here because only 2 fish did not enter the upper compartment.

Two-sided chi-square test was used to analyze initial preference (first choice) in BWPT. For general first choice regardless of treatment, hypothesized probability was 0.5. For treatment effects, first choice was analyzed by treatment in the chi-square test. Differences in cortisol between the fish that chose the black side compared to fish that chose the white side in the first choice were analyzed using the student *t*-test.

The association between NTDT and BWPT was analyzed using ANOVA. For the outcome variables from BWPT Entries_total_ and Entries_3min_, Entries_NTDT_ and treatment were fixed factors. For the other two outcome variables from the BWPT, namely Duration_total_ and Duration_3min_, Duration_NTDT_ and treatment were fixed factors. The interaction between the NTDT factor and treatment was not significant and was therefore excluded from both models. Correlation analysis was also carried out for further investigation of the association between NTDT and BWPT as this analysis is often applied in other studies and indicates the strength of the relationship between the two tests. The correlation between the number of entries into the white compartment in the BWPT and the number of entries into the upper compartment of the NTDT was analyzed by parametric (Pearson) correlation as the scatter plots were close to elliptical and the entries into the white compartment were close to being normally distributed. The correlation between the duration variables was analyzed by non-parametric (Spearman) correlation due to non-elliptical scatter plots and an evident deviation from the normal distribution in both variables.

## Results

### Cortisol Analysis

**Figure 2:**
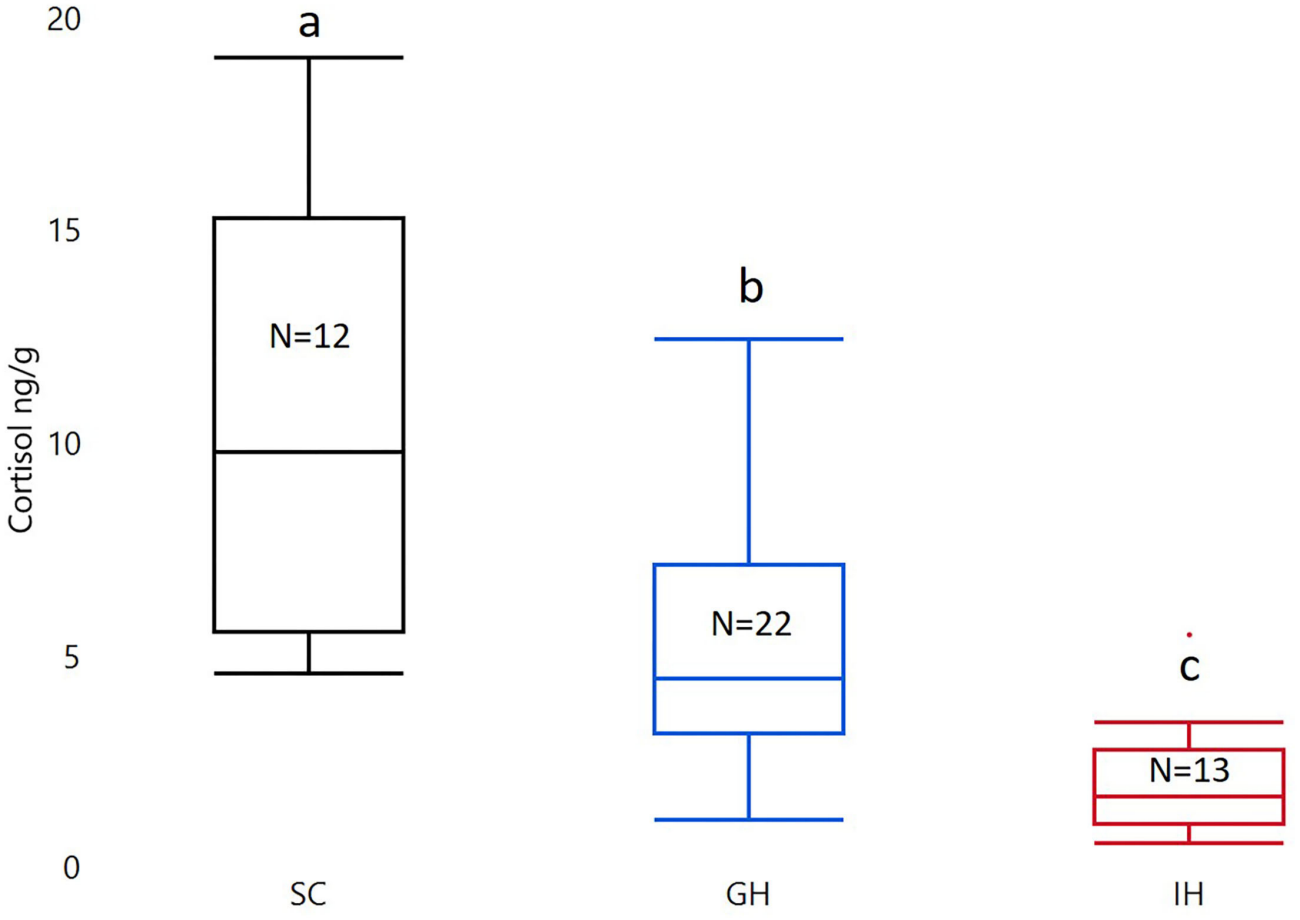
Box plots of untransformed whole-body cortisol in the two treatment groups (GH and IH) and fish from the initial stock (SC). The concentration of whole-body cortisol is given as ng/g. The material was analyzed with ANOVA on square root transformed cortisol with housing as a fixed factor. Significant group differences from post-test are marked with unique lowercase letters. SC, stock cortisol; GH, group housed; IH, individually housed; *N*, number of fish.

Housing had a significant effect on cortisol (F_2, 44_ = 23.13; *p* < 0.0001). The post-Tukey HSD test showed that individually housed fish (IHC; LS mean ± SEM; 1.37 ± 0.18) had significantly lower whole-body cortisol levels compared to group housed fish (GHC; 2.22 ± 0.14; *p* = 0.0016. See Figure 2). Both groups had significantly lower whole-body cortisol levels than fish from the rearing facility (SC; 3.15 ± 0.19; *p* < 0.0001, *p* = 0.0008; see Figure 2).

**Figure 3:**
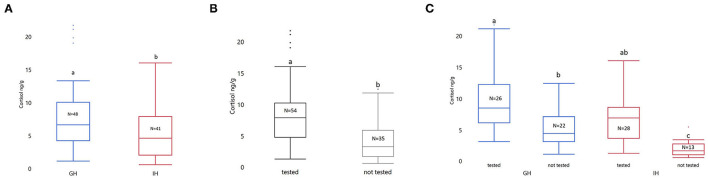
Box plots of untransformed whole-body cortisol sorted by **(A)** treatment, **(B)** tested vs. not tested, and **(C)** treatment by tested vs. not tested. The concentration of whole-body cortisol is given as ng/g. The data were analyzed with ANOVA on log transformed cortisol values. Housing, tested vs. not tested (±behavioral tests), and their interaction were included as fixed factors. Significant group differences from post-tests are marked with unique lowercase letters. Tested, fish tested in NTDT and BWPT; Not tested, not tested in any behavioral assays; GH, group housed; IH, individually housed; *N* = number of fish.

Behavioral testing and housing had a significant impact on whole-body cortisol (Housing: F_1, 85_ = 18.05; *p* < 0.0001. Behavioral testing: F_1, 85_ = 18.05; *p* < 0.0001. See Figures 3A,B). The interaction between housing and whether the fish underwent testing was significant (F_1, 85_ = 1.58; *p* = 0.0412). There was a significantly higher cortisol level in both group housed (GHC: LSmean ± SEM; 1.50 ± 0.13; GH-Behavior (GH-BWPT and GH-NTDT): 2.17 ± 0.12; *p* = 0.0017) and individually housed zebrafish (IHC: 0.54 ± 0.17; IH-Behavior (IH-BWPT and IH-NTDT): 1.77 ± 0.11; *p* < 0.0001) that were tested, compared to the GH and IH fish that had not been tested (see Figure 3C). Furthermore, the behavioral testing resulted in similar cortisol levels in the two housing groups (*p* = 0.4332). Fish that underwent behavioral testing (IH-BWPT, GH-BWPT, IH-NTDT, GH-NTDT; 1.97 ± 0.08) had higher cortisol than untested fish (IHC, GHC; 1,02 ± 0.11; post *t*-test *p* < 0.0001) regardless of housing treatment.

### Novel Tank-Diving Test

The zebrafish had a general preference for the lower compartment in the NTDT (mean ± SD Duration_total_ 118.78 ± 88.30; *p* < 0.0001),

Duration and entries were not affected by housing treatment (F_1, 48_ = 2.88, *P* = 0.0962), test order (F_1, 48_ = 0.11, *P* = 0.7425), or cortisol (F_1, 48_ = 1.12, *P* = 0.2953). There were no significant interactions between neither housing and first test (F_1, 48_ = 0.82, *p* = 0.3702) nor housing and cortisol (F_1, 48_ = 0.001, *p* = 0.9711).

The housing treatment groups did not differ in the latency to first enter the upper compartment in the NTDT (mean ± SD; GH 49.63 s ± 95.85; IH 41.11 s ± 59.99; S = 638, Z = −1.33, *p* = 0.1823).

### Black/White Preference Test

The zebrafish had a general preference for the black compartment in the BWPT (mean ± SD Duration_total_ 340.20 ± 160.41; *p* < 0.0001).

Neither housing, test order, cortisol, nor their interactions (housing × test order; housing × cortisol) had significant effects on duration_3min_ and Duration_total_ in the BWPT (see Table 2).

**Table 2:** The dependent variables and fixed effects for BWPT.

**Dependent variable**	**Effects**	***F*-value**	***p*-value**	**DF**
Entries_3min_	Treatment	0.7468	0.3918	1, 48
	Test order	0.0397	0.8430	1, 48
	Treatment × test order	4.6264	0.0365*	1, 48
	Cortisol	3.9026	0.0540	1, 48
	Treatment × cortisol	0.3611	0.5507	1, 48
Entries_total_	Treatment	1.8121	0.1846	1, 48
	Test order	0.1047	0.7477	1, 48
	Treatment × test order	5.7858	0.0201*	1, 48
	Cortisol	0.4961	0.4846	1, 48
	Treatment × cortisol	2.0416	0.1595	1, 48
Duration_3min_	Treatment	1.9129	0.1730	1, 48
	Test order	0.8738	0.3546	1, 48
	Treatment × test order	1.6790	0.2012	1, 48
	Cortisol	0.1739	0.6785	1, 48
	Treatment × cortisol	0.6134	0.4373	1, 48
Duration_total_	Treatment	0.4435	0.5086	1, 48
	Test order	1.8384	0.1815	1, 48
	Treatment × test order	1.9154	0.1728	1, 48
	Cortisol	0.0093	0.9238	1, 48
	Treatment × cortisol	0.7331	0.3961	1, 48

Model, dependent variable = treatment + test order + treatment × test order + cortisol + treatment × cortisol. Treatment, GH vs. IH; test order, BWPT vs. NTDT as the first behavioral assay; cortisol, whole-body cortisol. Every fixed effect is presented with its F-value, p-value, and degrees of freedom. P-values <0.05 are marked red and with *. P-values between 0.05 and 0.10 are marked orange.

**Figure 4:**
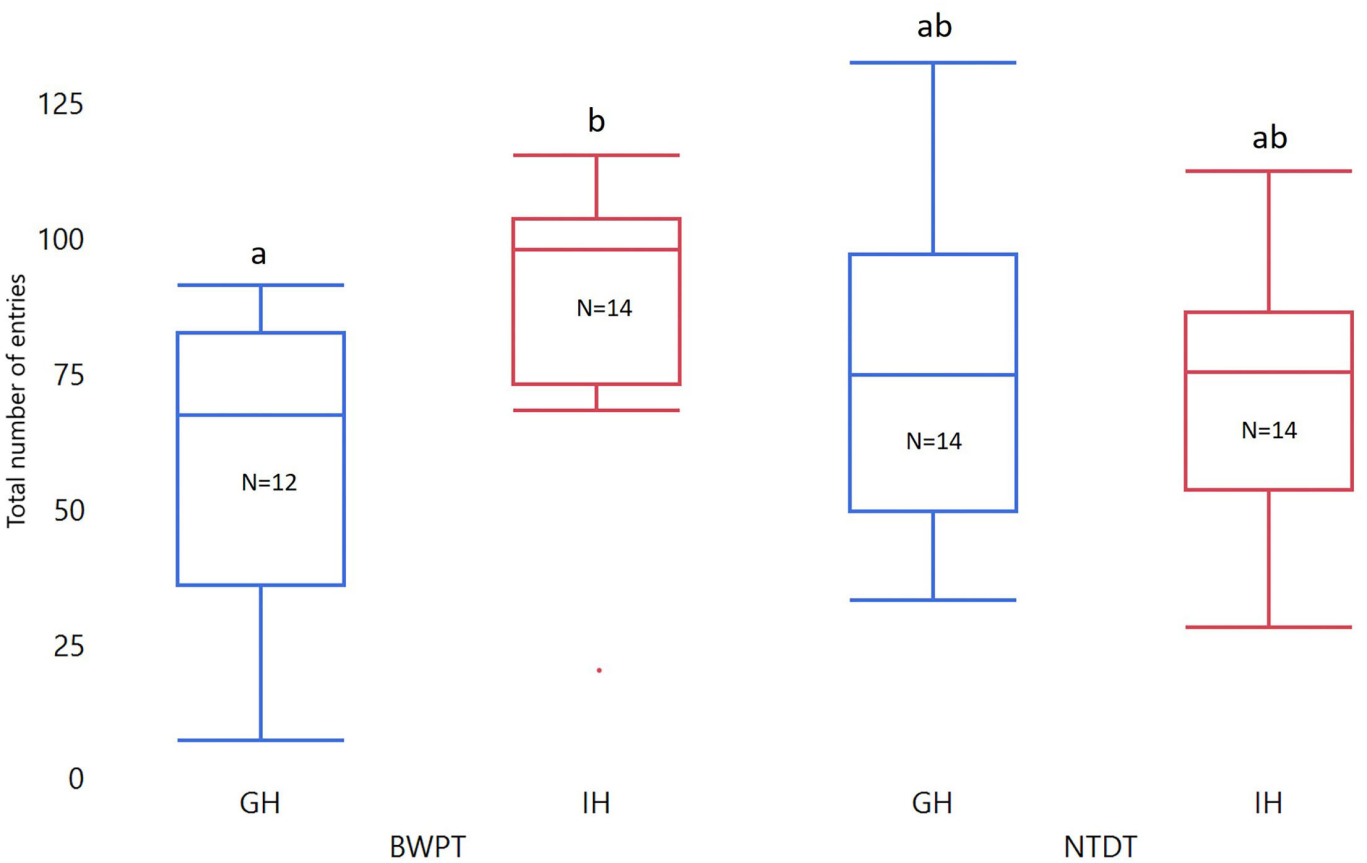
Box plots of total number of entries in BWPT. The data were analyzed with ANOVA. Treatment, test order, and whole-body cortisol concentration were entered as fixed factors, and the interaction between test order and treatment and treatment and cortisol were included. Significant group differences from post-tests are marked with unique lowercase letters. GH, group housed; IH, individually housed; BWPT, BWPT as the first behavioral test; NTDT, NTDT as the first behavioral test; *N*, number of fish.

Housing and test order did not affect number of entries to the white compartment in the BWPT. However, the interaction between housing and test order was significant for both Entries_3min_ and Entries_total_ (see Table 2). Two post-test comparisons were considered biologically relevant: individually housed zebrafish (IH) against group housed zebrafish (GH) with BWPT as their first behavioral test (IH-BWPT, GH-BWPT) and IH against GH with NTDT as their first behavioral test (IH-NTDT, GH-NTDT). The post-test did not find a significant difference between IH-BWPT and GH-BWPT in Entries_3min_ (IH-BWPT 19.0 ± 1.91; GH-BWPT 13.2 ± 2.04; student *t*-test, *p* = 0.0421; Bonferroni-corrected critical *p*-value = 0,025), but there was a difference in Entries_total_ (IH-BWPT 86.0 ± 7.37; GH-BWPT 57.9 ± 7.86; *p* = 0.0121; see Figure 4). IH-BWPT had higher numbers of entries than GH-BWPT (see Figure 4). The post-test failed to find a significant difference between IH and GH when the novel tank diving test had been their first test (Entries_3min_: IH-NTDT 14.6 ± 1.95; GH-NTDT 16.9 ± 1.93; *p* = 0.3935; Entries_total_: IH-NTDT 66.0 ± 7.54; GH-NTDT 73.2 ± 7.43; *p* = 0.4967).

Furthermore, there was a tendency toward an effect of cortisol on Entries_3min_, but not on Entries_total_ (see Table 2). The slope was negative (slope estimate = −0.433, SEM = 0.22, *t*-ratio −1.98, *p* = 0.054), and fish with higher cortisol levels tended to have lower numbers of entries to the white compartment during the first 3 min of the test.

Overall, there was a preference for the black side as the first choice in the BWPT (Likelihood ratio: Chi square 7.59, DF 1, *p* = 0.0059). There was no significant difference between GH and IH in the first choice (Likelihood ratio: Chi square 2.75, DF 1, *p* = 0.0972). Furthermore, there was no difference in cortisol between the fish that choose the black and the fish that chose the white side first (Mean ± SD Black 7.90 ± 5.06, White 9.43 ± 4.12 student *t*-test: *p* = 0.25).

### The Association Between the Novel Tank-Diving Test and the Black/White Preference Test

Entries_NTDT_, but not treatment, had a significant effect on both Entries_total_ (Entries_NTDT_: F_1, 51_ = 7.56, *p* = 0.0083; treatment: F_1, 51_ = 0.56, *p* = 0.4559) and Entries_3min_ (F_1, 51_ = 6.03, *p* = 0.0175; treatment: F_1, 51_ = 0.58, *p* = 0.4506) in BWPT. There was a positive association between Entries_NTDT_ and Entries_total_ (slope estimate = 0.60, SEM 0.22, t ratio 2.75, *p* = 0.0083) and Entries_3min_ (slope estimate = 0.14, SEM 0.06, t ratio 2.46, *p* = 0.0175). Fish that had higher numbers of entries into the upper half of the tank in the NTDT had higher numbers of entries into the white compartment in BWPT.

Neither Duration_NTDT_ nor treatment affected Duration_total_ in BWPT (Duration_NTDT_: F_1, 51_ = 0.36, *P* = 0.5522; treatment: F_1, 51_ = 0.69, *P* = 0.4103). Moreover, neither Duration_NTDT_ nor treatment affected Duration_3min_ in BWPT (Duration_NTDT_: F_1, 51_ = 0.27, *P* = 0.6087; treatment: F_1, 51_ = 2.85, *P* = 0.0975).

Significant positive correlations between Entries_NTDT_ and Entries_total_ in BWPT were found for the two treatment groups combined and within each treatment group (groups combined: correlation coefficient 0.40; *p* = 0.003; IH: 0.33; *p* = 0.08; GH 0.38; *p* = 0.05).

The correlation between the duration in the lower part of the tank in the NTDT and the duration in the black compartment in BWPT was neither significant for the treatment groups combined (Spearman's correlation coefficient = −0.06), nor analyzed separately (IH Spearman's correlation coefficient = −0.1, *p* = 0.63; GH Spearman's correlation coefficient = −0.20, *p* = 0.34).

## Discussion

We found that zebrafish housed in groups had higher whole-body cortisol levels than individually housed zebrafish (Figure 2). This finding is in accordance with other studies on the effect of social isolation on cortisol in zebrafish (6–8). Zebrafish are a highly social species which easily form shoals. It shows preference for conspecifics when exposed to visual cues of shoals (26–28) and visual cues of conspecifics have been used as reinforcement in associative learning studies (29, 30). In our experiment, both individually housed fish and group housed fish had markedly lower cortisol than fish from the rearing facility. The group housed zebrafish had a maximum density of 0.48 fish/L, which is considerably lower than in the rearing facility (5–7 fish/L). Ramsay et al. (9) exposed zebrafish to a density of 40 fish/L for 3 h and for 5 days, resulting in whole-body cortisol levels of 11.7 ng/g and 14.3 ng/g, respectively. These results were significantly different from those of the low-density group (0.25 fish/L) in the same experiment. The low-density group had whole-body cortisol of 3.2 ng/g, and the results were interpreted as a density evoked cortisol increase in the crowded group. In accordance with this study, the difference between our treatment groups and the fish from the rearing facility can be a result of group density. Why a species that prefers social contact with conspecifics shows higher baseline cortisol when group housed compared to individually housed is hard to explain. It may be a result of more interactions and stimulation due to group dynamics. Social hierarchies influence cortisol levels in zebrafish with higher plasma levels in subordinates (31), and hierarchies might have formed in the group housed fish in the current study. Among several ways of stimulation, zebrafish may be prone to chemical signals in the water, which was discussed in (32, 33). There, it was found that fish exposed to water in which other fish had been subjected to assumed stress exhibited significantly increased cortisol levels. This could indicate that the cortisol of zebrafish is influenced by chemical signal substances from conspecifics in their group.

Exposure to the two behavioral tests resulted in an acute elevation in whole-body cortisol (mean; GH 9.9 ng/g; IH 6.9 ng/g) compared to baseline cortisol (GHC 5.4 ng/g; IHC 2.0 ng/g) (Figure 3). This is as expected and indicates that both housing treatments left the fish with the capacity to respond physiologically to the acute stress that the behavioral tests were. It is also in accordance with other studies in zebrafish [after exposure to a novel tank: GH ~45 ng/g; IH ~25 ng/g (8); exposure to novel tank test: ~18 ng/g, exposure to light-dark test: 12 ng/g (24); group housed zebrafish tested individually in a novel tank: ~60 ng/g and not behavioral tested: ~10 ng/g (34)].

As in accordance with other studies, our zebrafish showed an initial and general preference (19, 20, 24) for the black compartment in the BWPT (scototaxis). We did not find any significant difference in total time spent in WC between the group housed and individually housed zebrafish. Housing conditions had an effect on the total number of entries to WC in the BWPT when BWPT was the first behavioral test, but not when the fish had already been tested in the NTDT (Figure 4). The exact mechanism remains unclear, but a possible explanation is that an acutely stressful event, e.g., the NTDT before the BWPT, may blunt the behavioral responses in the test. As such, the fact that individually housed fish showed more entries into the white compartment could indicate a bolder behavior in fish with lower whole-body cortisol at the start of the test. The blunting effect of the NTDT may be due to an increase in cortisol during the test that made the fish less prone to venturing into the white compartment. In support of this line of thought, we found that fish that had higher whole-body cortisol had a tendency toward fewer entries into the white compartment the first 3 min in the BWPT, but the effect was not consistent throughout the test. Our hypothesis was that anxious zebrafish would present with higher cortisol, show fewer entries, and lower duration time. Our results partly support this hypothesis, at least when considering the entries in the BWPT, but not the duration in either test. However, due to the ambiguous nature of the results, alternative explanations may be available. For example, we cannot disregard the possibility that group housed fish got more anxious due to a sudden social isolation during the test, which in turn may have affected the total number of entries into WC in BWPT.

In contrast to (7), who found that duration in the lower compartment in the NTDT was very sensitive to housing conditions, we did not detect any effect of housing on behaviors in the NTDT. The fish had a general preference for the lower compartment, but there was no difference between group housed and individually housed fish. An explaining factor might be that the test arena was divided in two, and not three, compartments, which could affect the test sensitivity [but see (35)]. Additionally, the crowd density in our group housed fish (0.48 fish/L), which might not be high enough to give a significant difference between the groups. Interestingly, (36) did not find any effects on duration between group housed and individually housed zebrafish in the NTDT with similar density as that used by Parker and colleagues.

Lindsey and Tropepe (37) found in their study that both acute isolation (1 h), chronic isolation (2 weeks), and exposure to a novel zebrafish group resulted in significant reduction in cortisol levels compared to group housed zebrafish. In the same study, there was no difference between the group reared zebrafish and zebrafish reared in isolation for 6 months. As both isolation and exposure to novel conspecifics are thought to be stressful events, the lack of cortisol increase is surprising. Schroeder and Sneddon (38) also described an unexpected cortisol pattern in their study on the effects of analgesic on the response to fin clipping. In the placebo group, the zebrafish that went through fin clipping (fin clipped: ~6 ng/g) had markedly lower reported cortisol than the not fin clipped placebo fish (sham control ~14 ng/g) that underwent the same procedure except for fin clipping. These results indicate that we do not fully understand how the zebrafish respond to stress.

However, our housing treatment resulted in a statistically significant difference between treatment groups in cortisol, but that difference in cortisol was not present after the behavioral assays (Figure 3). This indicates that both housing treatments left the fish with the capacity to respond physiologically to the acute stress that the behavioral tests were. Correlation between duration of time spent in the least favored compartment and number of entries into the least favored compartment between the tests was predicted if the two behavioral assays provoke a similar state of anxiety. There was a correlation between number of entries in NTDT and BWPT, but not as strong as reported in (24) (correlation coefficient: 0.75). The association was not affected by the housing conditions. Kysil et al. did also find a strong correlation (correlation coefficient 0.55) in duration between the two behavioral assays. Even though the fish in this study showed preference for the lower compartment in the NTDT and the black compartment in the BWPT, there was no association in duration between the two behavioral assays. The missing correlation in duration is unexpected as we predicted that duration would be a stable behavioral measurement of anxiety in the two behavioral assays. The lack of correlation questions duration of time spent in the least favorable compartment as a valid measurement of anxiety. As stated in (25), further validation of multiple behavioral anxiety assays in zebrafish is needed.

## Conclusion

We have shown that zebrafish housed in groups have higher whole-body cortisol than individually housed zebrafish. Our zebrafish had a general preference for the lower compartment in the NTDT and the black compartment in the BWPT. Individually housed zebrafish had a higher total number of entries to the white compartment in BWPT compared to group housed zebrafish when their first test was BWPT, but not when BWPT was their second behavioral test. Fish that had higher whole-body cortisol had a tendency toward fewer entries into the white compartment the first 3 min of the BWPT. There was no effect of treatment on the behaviors registered in the NTDT. There was a correlation between number of entries into the white compartment in the BWPT and the upper part of the tank in NTDT, but there was no correlation between the duration of time spent in those compartments in the two tests. This lack of correlation is interesting to note as it contradicts our expectation. We suggest that this indicates a need for further validation of these models. Moreover, the insufficient explanation of the cortisol levels in the treatment groups underlines a need for further research to deepen the knowledge of how to measure stress in zebrafish.

## Data Availability Statement

The raw data supporting the conclusions of this article will be made available by the authors, without undue reservation.

## Ethics Statement

The animal study was reviewed and approved by the Food Safety Authorities (FOTS ID 12271).

## Author Contributions

TO: conduction of the experiment, behavioral observation, and data analysis supervised by JN. TO: lab work and drafting the publication. All authors: research idea, experimental design, interpretation of data, and revision of publication. All authors contributed to the article and approved the submitted version.

## Funding

This study was funded by Department of Production Animal Clinical Sciences and Department of Paraclinical Sciences, NMBU.

## Conflict of Interest

The authors declare that the research was conducted in the absence of any commercial or financial relationships that could be construed as a potential conflict of interest.

## Publisher's Note

All claims expressed in this article are solely those of the authors and do not necessarily represent those of their affiliated organizations, or those of the publisher, the editors and the reviewers. Any product that may be evaluated in this article, or claim that may be made by its manufacturer, is not guaranteed or endorsed by the publisher.
